# Gene Polymorphisms Involved in Folate Metabolism and DNA Methylation with the Risk of Head and Neck Cancer

**DOI:** 10.31557/APJCP.2020.21.12.3751

**Published:** 2020-12

**Authors:** Tialfi Bergamin de Castro, Gabriela Helena Rodrigues-Fleming, Juliana Garcia de Oliveira-Cucolo, Jéssika Nunes Gomes da Silva, Fabia Pigatti Silva, Luiz Sérgio Raposo, José Victor Maniglia, Érika Cristina Pavarino, Lidia Maria Rebolho Batista Arantes, Ana Lívia Silva Galbiatti-Dias, Eny Maria Goloni-Bertollo

**Affiliations:** 1 *São Jose do Rio Preto Medical School (FAMERP), Molecular Biology Department, Genetic and Molecular Biology Research Unit (UPGEM), São José do Rio Preto, Brazil. *; 2 *São Jose do Rio Preto Medical School (FAMERP), Otorhinolaryngology and Head and Neck Surgery Department, São José do Rio Preto, Brazil. *

**Keywords:** Head and neck cancer, folate, DNA methylation, polymorphism

## Abstract

**Background: **Folate is essential for DNA synthesis, repair, and methylation. Polymorphisms in genes associated with folate metabolism may alter these processes and, consequently, modulate cancer development. **Aim:** We aimed to assess *DNMT3B -149C/T *(rs2424913), *DNMT3B -283T/C *(rs6087990), *DNMT3B -579G/T* (rs2424909), *DHFR 19-pb ins/del *(rs70991108), *SHMT1 1420C/T *(rs1979277), and *TYMS 28-bp* tandem repeat (rs34743033) polymorphisms with risk of head and neck cancer. **Methods:** A case-control study was conducted in 1,086 Brazilian individuals. Real-time and conventional polymerase chain reactions-PCR were performed for genotyping the polymorphisms. **Results:** The single nucleotide polymorphism (SNP), *DNMT3B -283T/C*, revealed a higher risk of head and neck squamous cell carcinoma (HNSCC) when compared with the C group in the codominant (p< 0.001), dominant (p<0.001), and overdominant (p= 0.001) models for T/C and C/C genotypes. *DNMT3B -149C/T *and *DNMT3B -579G/T *revealed no association between groups in any model. The DHFR 19-pb ins/del polymorphism protected against HNSCC development compared to the C group by the codominant (p< 0.001), dominant (p< 0.001), and overdominant (p< 0.001) models. In the TYMS, the 3R/3R genotype had a protective effect against HNSCC development compared with the C group by the recessive models (p= 0.009). In contrast, *SHMT1 1420 C/T* presented no association between the HNSCC and C groups. DHFR 19-pb ins/del polymorphisms protected against oral cavity cancer (p= 0.003), and only TYMS-28 3R/3R decreased the risk of tumor progression (p= 0.023). In the Kaplan–Meier curve, an association was found between DHFR ins/ins and *TYMS -28 3R* carriers with respect to relapse-free time; further, *DNMT3B -579 T* and *TYMS-28 2R/2R *carriers had longer survival times.** Conclusion: **DNMT3B -283T/C is associated with higher risk, whereas *DHFR 19-pb ins/del* and *TYMS 28 3R/3R *protect against head and neck cancer. We also highlighted the association of *TYMS 3R/3R* genotype carriers with relapse-free cancer protection and survival time.

## Introduction

Presently, head and neck cancer is considered the fifth most common type of cancer worldwide and is associated with a high mortality rate when diagnosed in advanced stages. In 2020, 834,860 new cases of this neoplasm were estimated (IARC, 2019). This cancer comprises a heterogeneous group of tumors that originate in lining epithelium squamous cells of the upper digestive tract, including the lip, oral cavity, nasal cavity, paranasal sinus, pharynx, and larynx (Chow, 2020).

Elderly males are mostly affected (Chow, 2020; Wang et al., 2017). The main risk factors include tobacco and alcohol consumption, viral infections, particularly with the Epstein-Barr virus and human papillomavirus subtypes 16 and 18, as well as deficiencies or imbalances in vitamins and micronutrients such as folate, vitamins A, C, and E, zinc, and selenium (Fan et al., 2017). Smoking and alcohol consumption together increase the risk of this cancer, particularly in the oral cavity and pharynx, as cigarettes have approximately 4,700 chemical substances, of which at least 50 are carcinogenic; frequent alcohol consumption prevents epithelial cells forming the protective barrier against external agents, thus permitting easy entry of carcinogens, thereby forming adducts of DNA that are not recognized during DNA replication and repair (Wang et al., 2017). 

Folate or vitamin B plays a pivotal role in one-carbon metabolism, and generally low levels can be associated with the absence of methylation and DNA repair, thereby promoting carcinogenesis (Pieroth et al., 2018). Thus, several functional gene polymorphisms linked to folate metabolism can cause an imbalance in methylation reactions, as well as free nucleotides for DNA synthesis and repair (Coppedè et al., 2019). 

In most tumor models, higher intake of folate might decrease the risk of cancer development, as observed in oropharyngeal (Galeone et al., 2015), esophageal (Ni et al., 2019; Tio et al., 2014), pancreatic (Liu et al., 2017), cervical (Zhou and Meng, 2016), and breast cancer (Zeng et al., 2020); however, an inverse association can be seen in some tumor models, such as head and neck (Fanidi et al., 2015) and prostate cancers (Tio et al., 2014).

Thus, considering the contradictory results and genetic heterogeneity of the Brazilian population, it is important to evaluate the role of certain folate polymorphisms for susceptibility to head and neck carcinogens. Here, we aimed to investigate the association of polymorphisms *DNMT3B -149C/T *(rs2424913), *DNMT3B -283T/C *(rs6087990), *DNMT3B -579G/T* (rs2424909), *DHFR 19-pb ins/del* (rs70991108), *SHMT1 1420C/T* (rs1979277), and *TYMS 28-bp* tandem repeat (rs34743033) with the risk of head and neck cancer. 

## Materials and Methods

*Ethics Statement*

The Research Ethics Committee of São José do Rio Preto Medical School (FAMERP) in São José do Rio Preto, São Paulo, Brazil, approved this study (Registration Number 013/2012), and written informed consent for the collection of biological material was obtained from all individuals.

*Subjects and Samples*

This was a case-control study. The case groups included 378 patients (337 men and 41 women; mean age, 59 years) with a confirmed histopathological diagnosis of cancer. The disease-free control group (C) included 708 Brazilian blood donors (517 men and 191 women; mean age, 47 years), without cancer diagnoses according to government guidelines for donated blood (http://bvsms.saude.gov.br/bvs/publicacoes/qualidade_sangue.pdf). DNA was obtained from the leukocytes from peripheral blood samples of all participants (case and control; total: 1086), and was genotyped for folate polymorphisms [*DNMT3B -149C>T* (rs2424913), *DNMT3B -283T>C *(rs6087990), *DNMT3B -579G>T* (rs2424909), *DHFR 19-pb ins/del *(rs70991108),* SHMT1 1420C>T* (rs1979277), and *TYMS 28-bp* tandem repeat (rs34743033)].

Individuals were classified as having early stage (T0, T1, and T2; N0 and M0) and advanced stage tumors (T3 and T4; N1, N2, N3, and M1) based on their tumor, node, and metastasis (TNM) staging. The case and control group participants were interviewed to obtain their demographic and lifestyle data. Those who smoked more than 100 cigarettes in their lifetime were considered as tobacco consumers and those who consumed four doses of alcohol per week were considered as alcohol consumers.

*Polymorphism Genotyping*

DNA was extracted from the peripheral blood following a previously published protocol (Miller et al., 1988) with certain modifications (using Ficoll-Paque™ PLUS to separate blood components). The *DNMT3B -149C/T *(rs2424913), *DNMT3B -283T/C* (rs6087990), and *SHMT1 1420C/T* (rs1979277) polymorphisms were detected via a real-time polymerase chain reaction (PCR) for allelic discrimination using the Step One PlusTM Real-Time PCR System equipment (Applied Biosystems, USA), whereas *DNMT3B -579G/T* (rs2424909) was detected via PCR-RFLP (PCR-Restriction Fragment Length Polymorphism), and allele-specific PCR was used to assess the *DHFR 19-pb ins/del *(rs70991108) and *TYMS 28-bp* tandem repeat (rs34743033). For both PCR techniques, the reaction solution contained the following: 1 × buffer, 15.3 μL of ultrapure H2O, 2.0 μL (0.10 μmol/L) of dNTPs, 0.5 μL (25 mmol/L) of MgCl2, 1.25 μL of each primer (25 mmol/L), 0.2 μL (1 U) of Taq DNA polymerase, and 200 ng of genomic DNA. The PCR amplification products were visualized on a 3% agarose gel (Invitrogen^®^) with ethidium bromide in the presence of a 100 bp molecular marker. 

*Statistical Analysis*

SNPStats software was used to calculate the odds ratios (ORs) and 95% confidence intervals (CIs) for the risk associations between polymorphisms and head and neck cancer (Solé et al., 2006). The multiple logistic regression models were adjusted for age, gender, drinking, and smoking habits. The effect of the polymorphisms was evaluated in the models as (1) codominant (heterozygous vs. wild-type homozygous and polymorphic homozygous vs. wild-type homozygous); (2) dominant (heterozygous + polymorphic homozygous vs. wild-type homozygous); (3) recessive (polymorphic homozygous vs. wild-type homozygous + heterozygous); (4) overdominant (heterozygous vs. wild-type homozygous + polymorphic homozygous); or (5) log-additive (polymorphic homozygous with 2 + heterozygous vs. wild-type homozygous). Statistical analyses were performed using GraphPad Prism version 5.0 (GraphPad Software, Inc., USA) and SNPstats (https://www.snpstats.net/start.htm) (Solé et al., 2006). The haplotype frequencies of DNMT3B were inferred using the Haploview program, version 4.0 (Barrett, 2009). A probability level (p) of < 0.05 was considered to indicate statistical significance. 

## Results

The *DNMT3B -149C>T *(rs2424913) was not in Hardy–Weinberg equilibrium (HWE) in the case group (p < 0.001); *DNMT3B -579G>T* (rs2424909), *DHFR 19-pb ins/del *(rs70991108), and *TYMS 28-bp *tandem repeat (rs34743033) were not in HWE in the case (p < 0.001, p = 0.032, p < 0.001, respectively) and control groups (p < 0.001, p = 0.026, and p < 0.001). The other polymorphisms complied with HWE in both the case and control groups.

The sample group presented a median age of 51 years, and male gender was prevalent in the case (89.0 %) and control (73.0 %) groups. The multiple logistic regression analysis that evaluated the sociodemographic characteristics and risk factors indicated that the male gender (OR = 3.03; 95% CI = 2.11–4.37; p < 0.001), age ≥ 51 years (OR = 13.16; 95% CI % = 9.66–17.94; p < 0.001), smoking habit (OR = 9.84; 95% CI = 7.04–13.77; p < 0.001), and alcohol consumption (OR = 3.18; 95% CI = 2.38–4.24; p < 0.001) were associated with greater susceptibility to the development of this neoplasia when compared to the control group .

*DNMT3B -283T/C* (rs6087990) SNP was associated with a higher risk of head and neck squamous cell carcinoma (HNSCC) on comparison with the C group in the codominant model for both T/C and C/C genotypes (OR = 2.58; 95% CI = 1.59–4.18; p < 0.001 and OR = 1.86; 1.08–3.22; p < 0.001, respectively) and dominant (OR = 2.32; 1.46–3.69; p < 0.001) and overdominant models (OR = 1.81; 95% CI = 1.26–2.59; p = 0.001). The other two SNPs evaluated in this gene, *DNMT3B -149C/T *(rs2424913) and *DNMT3B -579G/T* (rs2424909), presented no association between groups in any model evaluated (Table 1).

*DHFR 19-pb ins/del *(rs70991108) was associated with a protective effect against HNSCC development compared to the C group by the codominant (OR = 0.42; 95% CI = 0.27–0.65; p < 0.001), dominant (OR = 0.48; 95% CI = 0.32–0.73; p < 0.001), and overdominant models (OR = 0.48; 95% CI = 0.32–0.71; p < 0.001). Similar results were obtained for TYMS 28-bp tandem repeat (rs34743033), where the 3R/3R genotype was associated with a protective effect against HNSCC development when comparing the C group by the recessive model (OR = 0.51; 95% CI = 0.30–0.85; p = 0.009). In fact, SHMT1 1420C/T (rs1979277) revealed no association when compared to the HNSCC and C groups (Table 1).

All polymorphisms were investigated to evaluate their association with the primary site. Thus, the present study demonstrated an association between the *DNMT3B -283T/C SNP* and an increased risk of cancer in the oral cavity (OR = 2.436; 95% CI = 1.39–4.25; p = 0.001), pharynx (OR= 2.12; 95% CI = 1.02–4.41; p = 0.045), and larynx (OR = 2.16; 95% CI = 1.21–3.85; p = 0.006); however, DHFR 19-pb ins/del polymorphisms were associated with a protective effect against oral cavity cancer (OR= 0.53; 95% CI = 0.35–0.80; p = 0.003) (Table 2). 

With regards to the association of polymorphisms with the TNM staging system, only TYMS-28 3R/3R SNP was associated with decreased risk of tumor progression (OR = 0.55; 95% CI = 0.33–0.93; p = 0.023) (Table 3). 

 The DNMT3B haplotypes analyses revealed a higher frequency of the alleles DNMT3B -149C, 283C, and 579T in the case group than in the control group (33.0% and 25.4%, respectively; p < 0.001), as well as that of DNMT3B -149T, 283T, and 579G (34.3% and 29.4%, respectively; p = 0.031); however, contradictory results were observed for DNMT3B -149T, 283C, and 579T, (3.0% and 8.0%, respectively; p < 0.001), for DNMT3B -149T, 283T, and 579T haplotype (3.8% and 6.8%, respectively; p = 0.007), and for 149T, 283C, and 579G haplotypes (3.3% and 6.2% respectively; p = 0.007), which were more frequent in the control group than in the case group.

**Figure 1:**
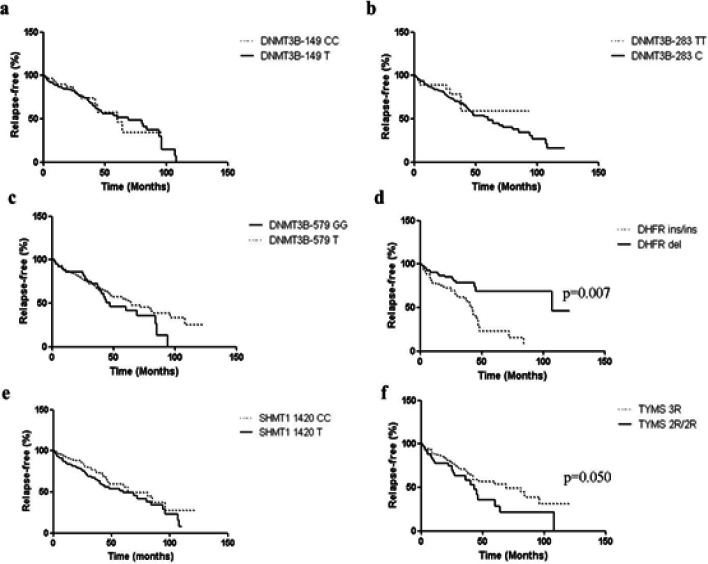
Kaplan–Meier Curves for Relapse-Free Time in Head and Neck Cancer Group. Comparison between (a) *DNMT3B* -149C/T: wild-type C/C and T polymorphic carriers, p = 0.741; (b) *DNMT3B* -283T/C: wild-type T/T and C polymorphic carriers, p = 0.479; (c) *DNMT3B* -579G/T: wild-type G/G and T polymorphic carriers, p = 0.413; (d) *DHFR* 19-pb ins/del: wild-type ins/ins and del polymorphic carriers, p = 0.007; (e) *SHMT1 *1420C/T: wild-type C/C and T polymorphic carriers, p = 0.068; and (f) *TYMS* 28-bp tandem repeat: 3R carriers and 2R/2R genotype, p = 0.050. Log-rank (Mantel–Cox) test

**Figure 2:**
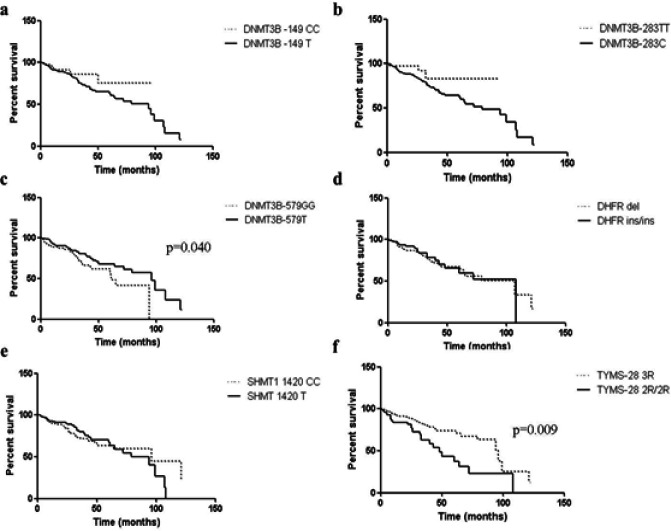
Kaplan–Meier Curves for Survival Time in Head and Neck Cancer Group. Comparison between (a) *DNMT3B* -149C/T: wild-type C/C and T polymorphic carriers, p = 0. 145; (b) *DNMT3B* -283T/C: wild-type T/T and C polymorphic carriers, p = 0.140; (c) *DNMT3B* -579G/T: wild-type G/G and T polymorphic carriers, p = 0.040; (d) *DHFR* 19-pb ins/del: wild-type ins/ins and del polymorphic carriers, p = 0.742; (e) *SHMT1* 1420C/T: wild-type C/C and T polymorphic carriers, p = 0.784; and (f) *TYMS* 28-bp tandem repeat: 3R carriers and 2R/2R genotype, p = 0.009. Log-rank (Mantel–Cox) test

In another statistical analysis, the Kaplan–Meier curve indicated the relapse-free and survival time in allele carriers and noncarriers (Figure 1 and 2, respectively). An association was observed in relation to the relapse-free time between DHFR del carriers (median = 100%) and DHFR ins/ins (median = 42%), p = 0.027, as well as, with the *TYMS -28 3R* carriers (median = 69%) and *TYMS-28 2R/2R* (median = 43%), p = 0.050. For the survival time, an association was observed between the *DNMT3B -579 G/G* (median = 61%) and *DNMT3B -579 T *carriers (median= 96%), p = 0.040, as well as between *TYMS-28 2R/2R *(median = 48%) and *TYMS-28 3R* carriers (median= 96%), p = 0.009. For the other polymorphisms analyzed, no relationship was observed between the relapse-free and survival times.

**Table 1.** Multiple Logistic Regression of DNMT3B -149 C/T, -283T/C, and -579G/T , DHFR, SHMT, and TYMS Polymorphisms in Individuals Free of Disease (Control-C) and Head and Neck Squamous Cell Carcinoma (HNSCC) Groups

Polymorphisms	Genotypes	Control, n (%)	Case, n (%)	OR (95% CI)
*DNMT3B* -149C/T (rs2424913)^a^				
Codominant	C/C	131 (23.8)	75 (22.8)	1
	C/T	287 (52.1)	168 (51.1)	1.01 (0.65 - 1.55)
	T/T	133 (24.1)	86 (26.1)	1.14 (0.69 - 1.87)
Dominant	C/C	131 (23.8)	75 (22.8)	1
	C/T-T/T	420 (76.2)	254 (77.2)	1.05 (0.70 - 1.58)
Recessive	C/C-CT	418 (75.9)	243 (73.9)	1
	T/T	133 (24.1)	86 (26.1)	1.13 (0.76 - 1.69)
Overdominant	C/C-T/T	264 (47.9)	161 (48.9)	1
	C/T	287 (52.1)	168 (51.1)	0.94 (0.66 - 1.34)
Additive	---	---	---	1.07 (0.83 - 1.37)
*DNMT3B *-283T/C (rs6087990)^b^				
Codominant	T/T	153 (26.3)	43 (13.6)	1
	T/C	278 (48.2)	194 (61.2)	2.58 (1.59 - 4.18)
	C/C	146 (25.3)	80 (25.2)	1.86 (1.08 - 3.22)
Dominant	T/T	153 (26.5)	43 (13.6)	1
	T/C-CC	424 (73.5)	274 (86.4)	2.32 (1.46 - 3.69)
Recessive	T/T-T/C	431 (74.7)	237 (74.8)	1
	C/C	146 (25.3)	80 (25.2)	0.93 (0.62 - 1.40)
Overdominant	T/T-C/C	299 (51.8)	123 (38.8)	1
	T/C	278 (48.2)	194 (61.2)	1.81 (1.26 - 2.59)
Additive	---	---	---	1.29 (0.99 -1.68)
*DNMT3B* -579G/T (rs2424909)^c^				
Codominant	G/G	193 (35.2)	110 (34.8)	1
	G/T	220 (40.1)	126 (39.9)	0.88 (0.58 - 1.34)
	T/T	135 (24.6)	80 (25.3)	0.88 (0.55 - 1.42)
Dominant	G/G	193 (35.2)	110 (34.8)	1
	G/T-T/T	355 (64.8)	206 (65.2)	0.88 (0.60 - 1.29)
Recessive	G/G-G/T	413 (75.4)	236 (74.7)	1
	T/T	135 (24.6)	80 (25.3)	0.95 (0.62 - 1.44)
Overdominant	G/G-T/T	328 (59.9)	190 (60.1)	1
	G/T	220 (40.1)	126 (39.9)	0.93 (0.64 - 1.34)
Additive	---	---	---	0.94 (0.74 - 1.18)
*DHFR 19*-pb ins/del (rs70991108)^d^				
Codominant	ins/ins	145 (28.0)	112 (37.3)	1
	ins/del	282 (54.4)	127 (42.3)	0.42 (0.27 - 0.65)
	del/del	91 (17.6)	61 (20.3)	0.70 (0.40 - 1.22)
Dominant	ins/ins	145 (28.0)	112 (37.3)	1
	ins/del-del/del	373 (72.0)	188 (62.7)	0.48 (0.32 - 0.73)
Recessive	ins/ins-ins/del	427 (82.4)	239 (79.7)	1
	del/del	91 (17.6)	61 (20.3)	1.19 (0.74 - 1.94)
Overdominant	ins/ins-del/del	236 (45.6)	173 (57.7)	1
	ins/del	282 (54.4)	127 (42.3)	0.48 (0.32 - 0.71)
Additive	---	---	---	0.76 (0.58 - 1.01)
*SHMT1* 1420C/T (rs1979277)^e^				
Codominant	C/C	280 (53.3)	166 (49.5)	1
	C/T	196 (37.3)	139 (41.5)	1.07 (0.73 - 1.56)
	T/T	49 (9.3)	30 (9.0)	0.68 (0.36 - 1.28)
Polymorphisms	Genotypes	Control, n (%)	Case, n (%)	OR (95% CI)
Dominant	C/C	280 (53.3)	166 (49.5)	1
	C/T-T/T	245 (46.7)	169 (50.5)	0.98 (0.68 - 1.40)
Recessive	C/C-C/T	476 (90.7)	305 (91)	1
	T/T	49 (9.3)	30 (9.0)	0.66 (0.36 - 1.21)
Overdominant	C/C-T/T	329 (62.7)	196 (58.5)	1
	C/T	196 (37.3)	139 (41.5)	1.14 (0.79 - 1.64)
Additive	---	---	---	0.91 (0.69 - 1.19)
TYMS 28-bp tandem repeat (rs34743033)f				
Codominant	2R/2R	123 (22.9)	85 (24.9)	1
	2R/3R	320 (59.5)	218 (63.9)	1.11 (0.71 - 1.72)
	3R/3R	95 (17.7)	38 (11.1)	0.55 (0.30 - 1.01)
Dominant	2R/2R	123 (22.9)	85 (24.9)	1
	2R/3R-3R/3R	415 (77.1)	256 (75.1)	0.96 (0.63 - 1.48)
Recessive	2R/2R-2R/3R	443 (82.3)	303 (88.9)	1
	3R/3R	95 (17.7)	38 (11.1)	0.51 (0.30 - 0.85)
Overdominant	2R/2R-3R/3R	218 (40.5)	123 (36.1)	1
	2R/3R	320 (59.5)	218 (63.9)	1.40 (0.96 - 2.03)
Additive	---	---	---	0.78 (0.58 - 1.05)
				

Odds Ratio (OR) adjusted for age, gender, alcohol, smoking habits, and polymorphisms; significant p values, p <0.05. ^a^Amplification was performed for 552 individuals in the control and 329 in the case groups; ^b^577 individuals in the control and 317 in the case groups; ^c^548 individuals in the control and in the case groups; ^d^518 individuals in the control and 300 in the case groups; ^e^525 individuals in the control and 335 in the case groups; and ^f^538 individuals in the control and 341 in the case groups.

**Table 2:** Association of the *DNMT3B* (-149C/T, -283T/C, -579G/T), *DHFR* 19-pb ins/del, *SHMT1* 1420C/T, and *TYMS* 28-bp Tandem Repeat Polymorphisms with the Primary Sites (Oral Cavity, Pharynx, and Larynx) in Head and Neck Cancer Group

Polymorphisms	Oral cavity		Pharynx		Larynx	
*DNMT3B* -149C/T (rs2424913)a	n (%)	OR (95 CI)	n (%)	OR (95 CI)	n (%)	OR (95 CI)
C/C	28 (22.0)	1	15 (21.0)	1	27 (24.0)	1
C/T-T/T	98 (78.0)	1.09 (0.68 -1.73)	50 (79.0)	1.16 (0.63 -2.12)	85 (76.0)	0.98 (0.61 - 1.58)
*DNMT3B* -283T/C (rs6087990)b						
T/ T	16 (13.0)	1	9 (15.0)	1	15 (14.0)	1
T/C-C/C	108 (87.0)	2.43 (1.39 - 4.25)	53 (85.0)	2.12 (1.02 - 4.41)	90 (86.0)	2.16 (1.21 - 3.85)
*DNMT3B* -579G/T (rs2424909)c						
G/G	45 (37.0)	1	20 (32.0)	1	35 (33.0)	1
G/T-T/T	78 (63.0)	0.94 (0.62 - 1.41)	43 (68.0)	1.16 (0.66 -2.04)	70 (67.0)	1.08 (0.69 - 1.69)
*DHFR *19-pb ins/del (rs70991108)d						
ins/ins	49 (42.0)	1	20 (36.0)	1	39 (37.0)	1
ins/del -del/del	67 (58.0)	0.53 (0.35 - 0.80)	35 (64.0)	0.68 (0.38 - 1.21)	66 (63.0)	0.65 (0.42 - 1.02)
*SHMT1* 1420C/T (rs1979277) e						
C/C	59 (45.0)	1	34 (51.0)	1	62 (55.0)	1
C/T-T/T	71 (55.0)	1.37 (0.93 - 2.02)	33 (49.0)	1.10 (0.66 - 1.84)	50 (45.0)	0.92 (0.61 - 1.38)
*TYMS* 28-bp tandem repeat(rs34743033)f						
2R/2R	38 (28.0)	1	11 (16.0)	1	26 (23.0)	1
2R/3R-3R/3R	100 (72.0)	0.78 (0.51 - 1.19)	56 (84.0)	1.50 (0.76 - 2.97)	85 (77.0)	0.96 (0.59 - 1.57)

Odds Ratio (OR) adjusted for age, gender, smoking habit and alcohol consumption. ^a^Amplification was possible for 126, 65 and 112 patients for oral cavity, pharynx and larynx, respectively; ^b^124, 62 and 105 patients for oral cavity, pharynx and larynx, respectively; ^c^123, 63 and 105 patients for oral cavity, pharynx and larynx, respectively; ^d^116, 55 and 105 patients for oral cavity, pharynx and larynx, respectively; ^e^130, 67 and 112 patients for oral cavity, pharynx and larynx, respectively; ^f^138, 67 and 111 patients for oral cavity, pharynx and larynx, respectively.

**Table 3:** Association of the *DNMT3B* (-149C/T, -283T/C, -579G/T), *DHFR* 19-pb ins/del, *SHMT1 *1420C/T, and *TYMS* 28-bp Tandem Repeat Polymorphisms with Tumor, Nodes, and Metastasis (TNM) Staging System in Patients with Head and Neck Cancer

Polymorphisms	Tumor progression (TNM)		
	Early	Advanced	OR (95 CI)
*DNMT3B* -149C/T (rs2424913)^a^			
C/C	40 (22.9)	32 (23.2)	1
C/T-T/T	135 (77.1)	106 (76.8)	0.99 (0.57 - 1.69)
*DNMT3B* -283T/C (rs6087990)^b^			
T/T	17 (10.2)	25 (18.2)	1
T/C-C/C	149 (89.8)	112 (81.8)	0.55 (0.28 - 1.07)
*DNMT3B* -579G/T (rs2424909)^c^			
G/G	62 (37.1)	44 (33.1)	1
G/T-T/T	105 (62.9)	89 (66.9)	1.18 (0.73 -1.93)
*DHFR *19-pb ins/del (rs70991108)^d^			
ins/ins	67 (40.4)	40 (32.5)	1
ins/del-del/del	99 (59.6)	83 (67.5)	1.50 (0.91 - 2.46)
*SHMT1* 1420 C/T (rs1979277)^e^			
C/C	89 (50.0)	73 (51.8)	1
C/T-T/T	89 (50.0)	68 (48.2)	0.92 (0.59 -1.45)

Odds Ratio (OR) adjusted for age, gender, smoking habit and alcohol consumption. ^a^313 patients; ^b^303 patients; ^c^300 patients; ^d^362 patients; ^e^319 patients; ^f^326patients.

## Discussion

In this study, we found that the sociodemographic characteristics and risk factors indicated that the male gender, advanced age, smoking, and alcohol consumption were associated with greater susceptibility to cancer development when compared to the control group. Head and neck cancer has a multifactorial etiology and involves various risk factors. 

In most countries, the rate at which men are affected is approximately two to five times higher than that of women, and this difference is presumably related to the adverse effects of carcinogens such as alcohol and tobacco, more commonly observed among men. Excessive consumption of carcinogens can affect nutrient absorption by the intestine, causing major nutritional deficiencies, and can modify the metabolic pathways, such as that of folate, which is essential for purine and pyrimidine synthesis and DNA methylation (Rettig and D’Souza, 2015). Moreover, the consumption of two or more cigarette packs along with four or more alcoholic drinks per day increases the risk of developing this neoplasia by 35-fold (Canova et al., 2010). 

Furthermore, the elderly are prone to cancer risk, with a median diagnosis age of approximately 60–70, which is higher than the median age considered at risk in this study (Cohen et al., 2018). 

Numerous studies have explored the association of *DNMT3B* polymorphisms and cancer risk but yielded conflicting results. One meta-analysis suggests that *DNMT3B -283T/C* and *DNMT3B -579G/T* may play a protective role against different types of cancers. Moreover, in the subgroup analysis, *DNMT3B -579G/T* appeared to contribute to decreased risk of lung and colorectal cancer, whereas *DNMT3B -149C/T *was associated with a decreased risk of head and neck cancer (Zhang et al., 2015). Another systematic evaluation of cancer risk demonstrated that *DNMT3B -149C/T, DNMT3B* -283T/C, and *DNMT3B -579G/T* polymorphisms were observed as protective factors against cancer in the Asian population (Duan et al., 2015); however, in our study, the *DNMT3B -149C/T *and *-579G/T SNPs *did not contribute to the risk of head and neck cancer, whereas *DNMT3B -283T/C* might be a risk factor for head and neck carcinogenesis. Li et al., (2016) demonstrated that *DNMT3B -283T/C* (rs6087990) has a potential effect on gastric cancer initiation.

We found that *DHFR 19-pb ins/del* (rs70991108) and *TYMS 28-bp* tandem repeat (rs34743033) polymorphisms have a protective effect against head and neck cancer. Our study is the first to evaluate the association between a DHFR 19pb deletion polymorphism and the risk of this neoplasia. Evaluation in other cancer types reveals different results. Jokic et al., (2011) and Liu et al., (2013) demonstrated that DHFR deletion was not associated with colon cancer risk. In contrast, Xu et al., (2007) reported an increased breast cancer risk in women with this gene deletion. Corroborating our findings, one study evaluated the *DHFR 19-pb ins/del *(rs70991108) polymorphism in mother/child dyads in acute lymphoblastic leukemia onset-latency and demonstrated a good prognosis for carrier patients with homozygous deletion (Tisato et al., 2019).

The role of *TYMS 28-bp* tandem repeat polymorphisms was not associated with acute lymphoblastic leukemia and lung cancer risk development (Oosterom et al., 2018; Stanisławska-Sachadyn et al. 2019); however, a study in the Brazilian population revealed increased risk association between the 2R/2R and 2R/3R variants in sporadic and hereditary breast cancer development, which was not in accordance with the results of our study (da Silva Nogueira et al., 2012).

In our study, the *SHMT1 *1420C/T (rs1979277) polymorphism had no association with head and neck cancer risk; however, it was associated with tumor progression. The human* SHMT1* gene is located at chromosome 17p11.2; the cytosolic isoform and its coenzyme, vitamin B6, catalyze the reversible conversion of serine and tetrahydrofolate to glycine and 5,10-methylene tetrahydrofolate that provide one-carbon units during pyrimidine and purine syntheses (Coppedè et al., 2019). A meta-analysis revealed that no association was found between *SHMT1 1420C>T* (rs1979277) and the overall risk of cancer; however, in the subgroup analysis, significant associations with a protective effect were found in colorectal cancer and in the Asian population (Wang et al., 2014). 

A preliminary study by our research group also found no risk association with head and neck cancer in the Brazilian population (Succi et al., 2014), which was in accordance with the results obtained for gastric cancer (Kim et al., 2016), colorectal cancer (Komlósi et al., 2010), non-Hodgkin lymphoma (Skibola et al., 2004), lung cancer (Wang et al., 2014), and breast cancer (Lissowska et al., 2007). According to Cheng et al., (2008), mutations in SHMT1 leading to an aberrant protein can be compensated for by the wild-type *SHMT2* gene that encodes an isoform with the same function. 

This is the first study to demonstrate an association between survival and relapse-free times. Individuals with wild-type genotype, *DNMT3B -579 G/G*, have longer survival times in relation to *DNMT3B -579 T* carriers, and this is also true for *TYMS*-3R carriers with respect to the *TYMS-*2R/2R genotype. Moreover, this last polymorphism protects *TYMS*-3R/3R genotype carriers against cancer recurrence. In vitro and in vivo studies have been carried out to verify the functional consequences of the variable number of tandem repeat polymorphism. In general, the *TYMS* alleles contain two or three copies of repeats (*2R* and *3R*), and the *TS* genes with triple sequence have higher expression levels than those with double sequence, and thus have transcriptional activity with the 3R sequence, which can be three to four times greater than that with the 2R carriers (Gusella and Padrini, 2007). 

A previous study that involved a smaller number of head and neck cancer patients evaluated survival and relapse-free time for the* DNMT3B 46359C/T* and* SHMT1 1420C/T* polymorphisms, but failed to demonstrate any statistically significant association between the wild-type and polymorphic carriers (Succi et al., 2014). In the present study, no association was observed between the *SHMT1 1420C/T* polymorphism and survival time and relapse-free cancer.

Thus, our findings reveal that *DNMT3B -283T/C* is associated with cancer risk, whereas *DHFR 19-pb ins/del* and *TYMS 28-bp* tandem repeat polymorphisms have a protective effect against head and neck cancer in relation to that in the control group. Furthermore, we highlight the association of the* TYMS 3R/3R* genotype with relapse-free cancer protection and the increased survival time of these patients in relation to those with the TYMS 2R allele.
